# Double-layer polarization-independent achromatic metasurface array for optical fiber bundle coupling in microendoscope

**DOI:** 10.1038/s41598-022-24785-3

**Published:** 2022-11-28

**Authors:** Yan Sun, Chang Wang, Shuhang Zheng, Xiao Tao, Xinyu Liu, Yong Li, Fei Wu, Zhenrong Zheng

**Affiliations:** 1 State Key Laboratory of Modern Optical Instrumentation, College of Optical Science and Engineering, Zhejiang University, Hangzhou, 310027 China; 2 Intelligent Optics and Photonics Research Center, Jiaxing Research Institute, Zhejiang University, Jiaxing, 314000 China; 3 Shanghai Aerospace Control Technology Institute, Shanghai, 201109 China; 4 Beijing LLVision Technology Co., Ltd., Room 301, Building B12C, No. 10 Jiuxianqiao Rd, Chaoyang District, Beijing, 10015 China

**Keywords:** Imaging and sensing, Metamaterials

## Abstract

Optical fiber bundle-based microendoscope, which is significant in clinical diagnosis and industrial detection, calls for miniaturization of the probe and high-resolution observation. Here, we propose a double-layer metasurface array borrowing the structures of insect compound eyes to meet both requirements instead of traditional optical components. Each unit in the array aims for an incident field of view, focusing light at the center of the fiber end face with no chromatic aberration at the wavelengths of 470 nm, 530 nm and 630 nm. The metasurface array is composed of a series of isotropic TiO_2_ nanopillars which are special selected after considering resonance mode and angular dispersion characteristics, etched on both sides of a silica substrate, with the individual functions of deflecting and focusing. In image space, numerical aperture (NA) is 0.287 and the particular layout of two layers achieve zero telecentricity theoretically, which meet the requirements of optical fiber bundle coupling. A unit for incident angle of 20° is shown to validate our design approach numerically, which obtains a focused spot close to the diffraction limit. The compact and ultrathin metasurface could greatly reduce the size of the probe in optical fiber bundle based microendoscope while ensuring the imaging quality.

## Introduction

Microendoscope plays an important role in both industry and medical fields, and provides a solution for obtaining image information from remote or hard-to-reach places^1^. For instance, in early cancer screening, the microendoscope system can display vivo imaging results in real time, avoiding the time-consuming and complicated operation of biopsy tissue for pathological section imaging^2,3^. Due to the extremely thin, soft and bendable characteristic of optical fiber bundle (OFB), tube microendoscope based on OFB is widely used^4^. For fiber microendoscope system, miniaturization of the probe and high resolution are two important matters, and objectives that couple images into OFB determine the imaging quality and the volume of the probe^5,6^. Each fiber arranges neatly in the bundle as an image point. In consideration of the special characteristics of OFB, the image coupling needs to meet the requirements of wide field of view (FOV), high telecentricity in image space and zero chromatic aberration. Traditional optical systems have to use multiple spherical lenses to achieve the above design requirements, which is undoubtedly difficult to further miniaturization^7^.

With the continuous development of precise machining technology, such as electronic beam etching^8,9^ and nanoimprint lithography technology^10^, dielectric metasurfaces provide a new avenue for replacing traditional bulky optical components by its superior ability of controlling amplitude, phase and polarization of electromagnetic waves. In recent years, more and more excellent studies on metasurfaces have been proposed in various fields, for example, planar lens^11,12^, vortex beam generators^13,14^, holographic displays^15,16^ and so on. Besides, in order to achieve miniaturization of devices in the area of signal processing, biological sensing and imaging, Pro. Cusano group integrated a phase-gradient plasmonic metasurface on the fiber tip to enable advanced wavefront manipulations^17^, and all-dielectric fluorescence enhancing metasurfaces on the end-face of a multimode fiber to realize lab-on-fiber optrodes^18^. In image systems, metalenses applied on eliminating various monochromatic aberrations, such as spherical aberration and coma^19^, have been devised by optimizing the phase profiles^20^. However, due to the various responses of metasurfaces to electromagnetic waves with different frequencies, it is still a major challenge to completely overcome chromatic aberrations^21^.

In this paper, we propose a double-layer polarization-independent achromatic metasurface array (PIAMA) resembling compound eye of insects to couple the image into the OFB in microendoscope. The diameter of each unit in PIAMA 30 μm and numerical aperture (NA) in image space is 0.287. The metasurface array should be fixed at a distance of 50 μm in front of the end face of OFB, which transmits the image point to the other end. The number and arrangement of the units in PIAMA can be flexibly designed according to that of fibers in the OFB. The PIAMA can be manufactured by etching isotropic titanium dioxide (TiO_2_) nanopillars on two sides of a silica substrate to realize the achromatic phase profiles at the wavelengths of 470 nm, 530 nm and 630 nm. We provide a way to design each unit in PIAMA and the characteristics of unit aimed for 20° incidence is theoretically investigated and numerically verified by finite difference time domain (FDTD) method. The results well illustrate the deflection and focus functions of the two layers. Compared to optical refractive lenses^22^ which are usually complex in structure and bulky in volume or gradient refractive index (GRIN) lens^23^ which is difficult to correct the off-axis aberration at the edge of the imaging field, our double-layer PIAMA can ensure image quality while reducing size, when used in microendoscopic probes. Furthermore, although our PIAMA has a double-layer structure, the position of nanopillars from both layers are not in one-to-one relationship, so there is no need to consider the errors caused by the misalignment problem in actual manufacture.

## System and method

### Structure of the PIAMA

**Figure 1:**
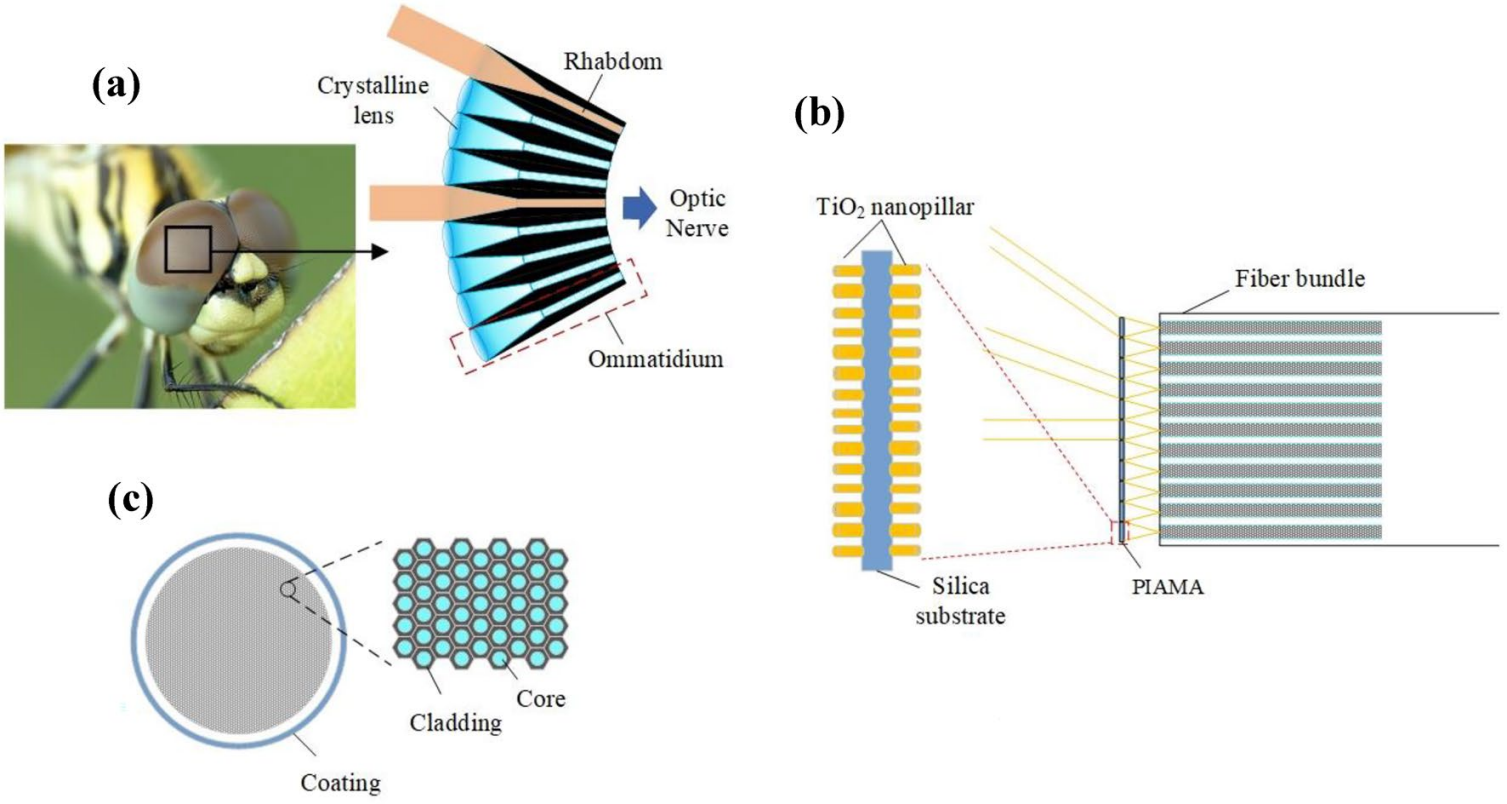
(**a**) Schematic of natural apposition compound eyes^31^. (**b**) Schematic of the fiber bundles arranged in a hexagonal shape. (**c**) Schematic of the fiber bundle coupled objective, PIAMA, proposed in this paper.

In natural creatures, apposition compound eyes of insects which possess a large view angle and the ability of detecting fast movement, contain numerous identical and repeating visual units called ommatidium, consisting of a single crystalline lens and rhabdom as shown in Fig. 1a, and the light through each ommatidium is received by only one photoreceptor^24^. We find that each photoreceptor cell is analogous to a pixel of sensor to receive image from sub-FOVs by one crystalline lens^25^. For the OFB used to transmit entire image, each optical fiber transmits a separate image point. Therefore, it is a good idea to design the coupling objective of the fiber microendoscope by referring to the structure of insect apposition compound eye.

Usually, both crystalline lens and its corresponding photoreceptor cell of apposition compound eyes are arranged as a spherical image surface, allowing each ommatidium to aim at different directions. However, this structure produces a curved-image-surface^26^, making image difficult to be collected by the end face of the OFB. Traditional artificial compound eyes are usually made up of micro-lens arrays, and solutions such as adding an intermediate system or utilizing optical prisms to deflect light beams^27^, or introducing multiple layers of micro-lens arrays^28^, are often considered in order to obtain planar images, undoubtedly increasing the system complexity.

In this work, we employ planar optical component, PIAMA, to match the design requirements of the OFB. Each unit in PIAMA is a metalens with a different off-axis angle incidence, and is designed to converge light from particular incident angle into a specific fiber. We choose a series of centrosymmetric TiO_2_ nanopillars which are polarization-independent on the silica substrate. In the visible range, TiO_2_ is an ideal material because of its high refractive index and nearly zero imaginary parts, which means negligible absorption^29^. From Palik’s handbook^30^, the real parts in refractive index of TiO_2_ are 2.5183, 2.4511 and 2.3903, and imaginary parts are 3.71 × 10^−6^, 1.33 × 10^–7^ and 2.19 × 10^–9^ at the wavelengths of 470 nm, 530 nm and 630 nm, respectively. Each nanopillar can be regarded as a cross-sectional truncated waveguide supporting multiple Fabry–Perot resonances with a low-quality factor. Because of the high refractive index contrast between TiO_2_ and air, the light energy is strongly confined inside each nanopillar. In addition, the coupling effect between neighboring nanopillars is extremely weak and can be ignored. The effective propagation constant of nanopillars is determined by its geometric parameter and the frequency of the incident electromagnetic wave, thereby generating different phase delays to achieve the purpose of wavefront manipulation.

The detailed layout of PIAMA used for OFB coupling is shown in Fig. 1b, which consists of two layers with separately different functions and purposes. The first layer is a light deflector with correcting chromatic aberration, allowing light of different incident angles to enter the second layer vertically. Meanwhile, units of the second layer are achromatic and aplanatic metalenses, which are exactly identical and match the NA of the fiber with theoretically zero telecentricity in image space. In PIAMA, each unit corresponds to one optical fiber in the OFB. Therefore, the amount and arrangement of units in PIAMA are consistent with those of OFB. For example, if optical fibers in OFB are arranged in hexagonal claddings as shown in Fig. 1c, units should also be arranged in hexagon. Here, a unit for incident angle of 20° is verified in our design approach for each layer of metasurfaces.

### The design of the deflecting layer

Light deflector manipulates the direction of transmissive electromagnetic waves in accordance with the generalized Snell’s law of refraction^32^. For different wavelengths, the different phase mutation needs to be introduced at the interface and can be written as:

1 $$ \phi \;(x,\;\lambda_{d} ) = \frac{2\pi }{{\lambda_{d} }} \cdot (n_{{\text{ t}}} \sin \;\theta_{t} - n_{{\text{ i}}} \sin \;\theta_{i} ) \cdot x, $$

where *n*_i_ and *θ*_i_ are the refractive index of the incident media and angle of incidence, respectively; *n*_t_ and *θ*_t_ are the refractive index of the outgoing media and angle of refraction, respectively; *λ*_d_ is the free-space wavelength and *x* is the position on the metasurfaces in Cartesian coordinate system. In this work, light is expected to be perpendicular to the second layer, so the exit angle *θ*_t_ of the first layer is 0°, and the light is incident from the air, which means *n*_i_ = 1. Therefore, the phase profile of the first layer can be calculated by

2 $$ \phi \;(x,\;\lambda_{d} ) = - \frac{2\pi }{{\lambda_{d} }} \cdot (\sin \;\theta_{i} ) \cdot x, $$

which is just related with incident angle, wavelength and position.

**Figure 2:**
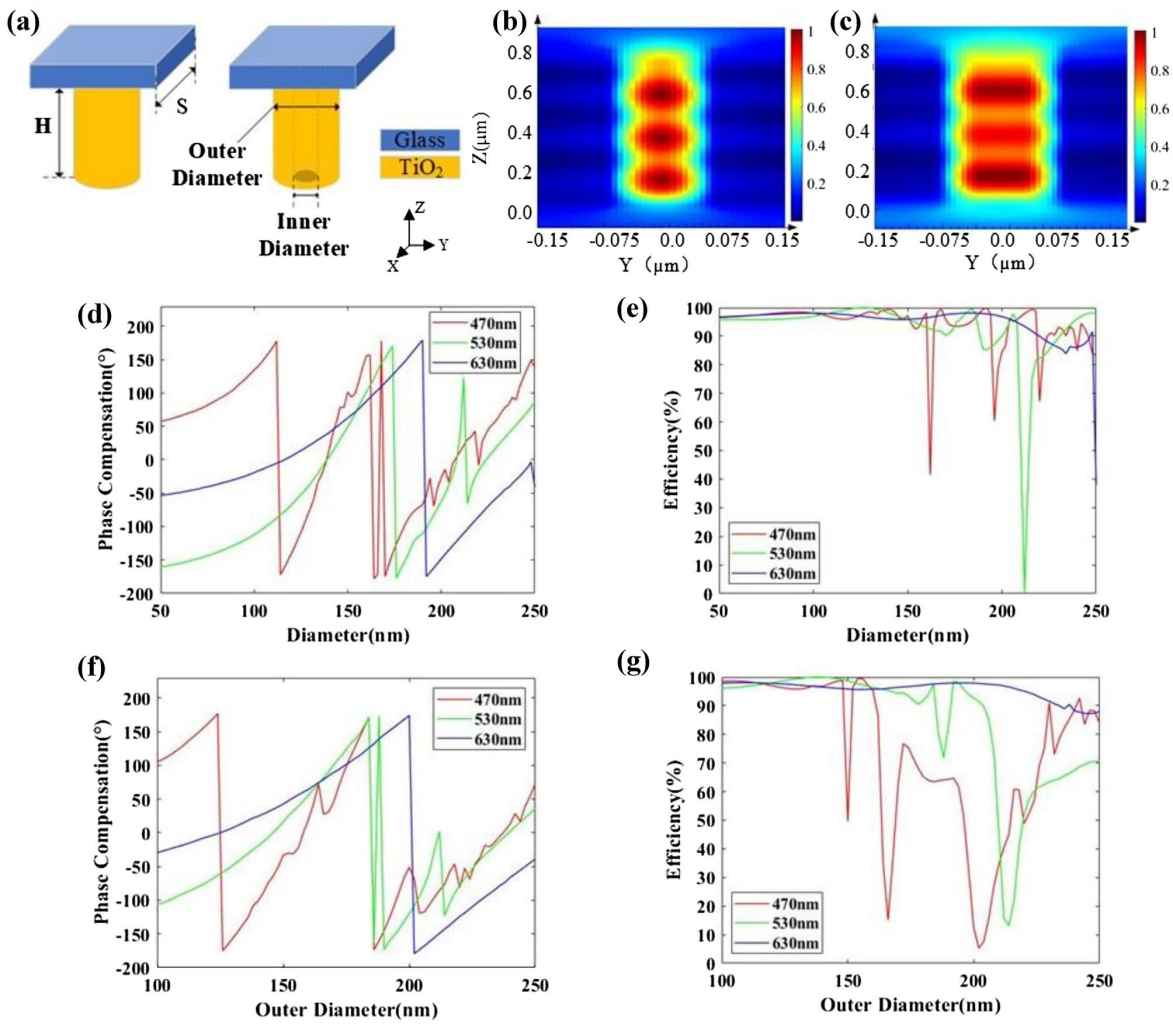
(**a**) Schematic of the nanostructure in deflecting layer. The TiO_2_ nanopillar with a height of 800 nm is placed under a glass substrate. The period of the nanostructure is 300 nm. (**b**,**c**) Magnetic field intensity distribution of cylindroid nanopillar and annular nanopillar at the wavelengths of 530 nm, respectively. (**d**,**e**) Phase compensation and transmission efficiency with the change of diameter of cylindroid nanopillars at the wavelengths of 470 nm, 530 nm and 630 nm, respectively. (**f**,**g**) Phase compensation and transmission efficiency with the change of outer diameter and the inner diameter of 50 nm of annular nanopillars at the wavelengths of 470 nm, 530 nm and 630 nm, respectively.

To realize the metasurfaces for achromatic light deflecting, we choose two kinds of isotropic nanostructures, cylindroid nanopillar and annular nanopillar as shown in Fig. 2a. Electromagnetic field response of nanostructures is quantitatively calculated in the commercial software package (Lumerical FDTD Solutions), where the height and period are set as 800 nm and 300 nm, respectively. To illustrate the effectiveness of the selection of structural parameters, the magnetic field intensity distribution of a cylindroid nanopillar with diameter of 140 nm and an annular nanopillar with inner diameter of 50 nm as well as outer diameter of 150 nm are shown in Fig. 2b,c, respectively, indicating the power mainly distributes inside the nanopillars. All the nanopillars chosen in PIAMA own the similar electromagnetic resonance mode with the energy strongly confined inside itself and the weak coupling effect between the neighboring nanopillars. Different phase compensation ranging from 0 to 2π are provided by changing the diameter of cylindroid nanopillars and the inner/outer diameters of annular nanopillars. The simulated phase compensation and the transmission efficiency of cylindroid nanopillars and annular nanopillars with inner diameter of 50 nm are shown in Fig. 2d–g, respectively. The design theory of metasurfaces is based on the Huygens Principle^33^, so the phase, amplitude and polarization of each secondary wavelength can be changed by placing a layer of resonators with subwavelength structures on the interface. In many researches, only the resonance excited by the electromagnetic wave of normal incidence is considered, but in fact, the resonance of the nanopillars may change with the incident angle of the electromagnetic wave. For the first layer metasurface, the angular dispersion of the nanopillars should be considered when light is incident at different angles^34^.

**Figure. 3:**
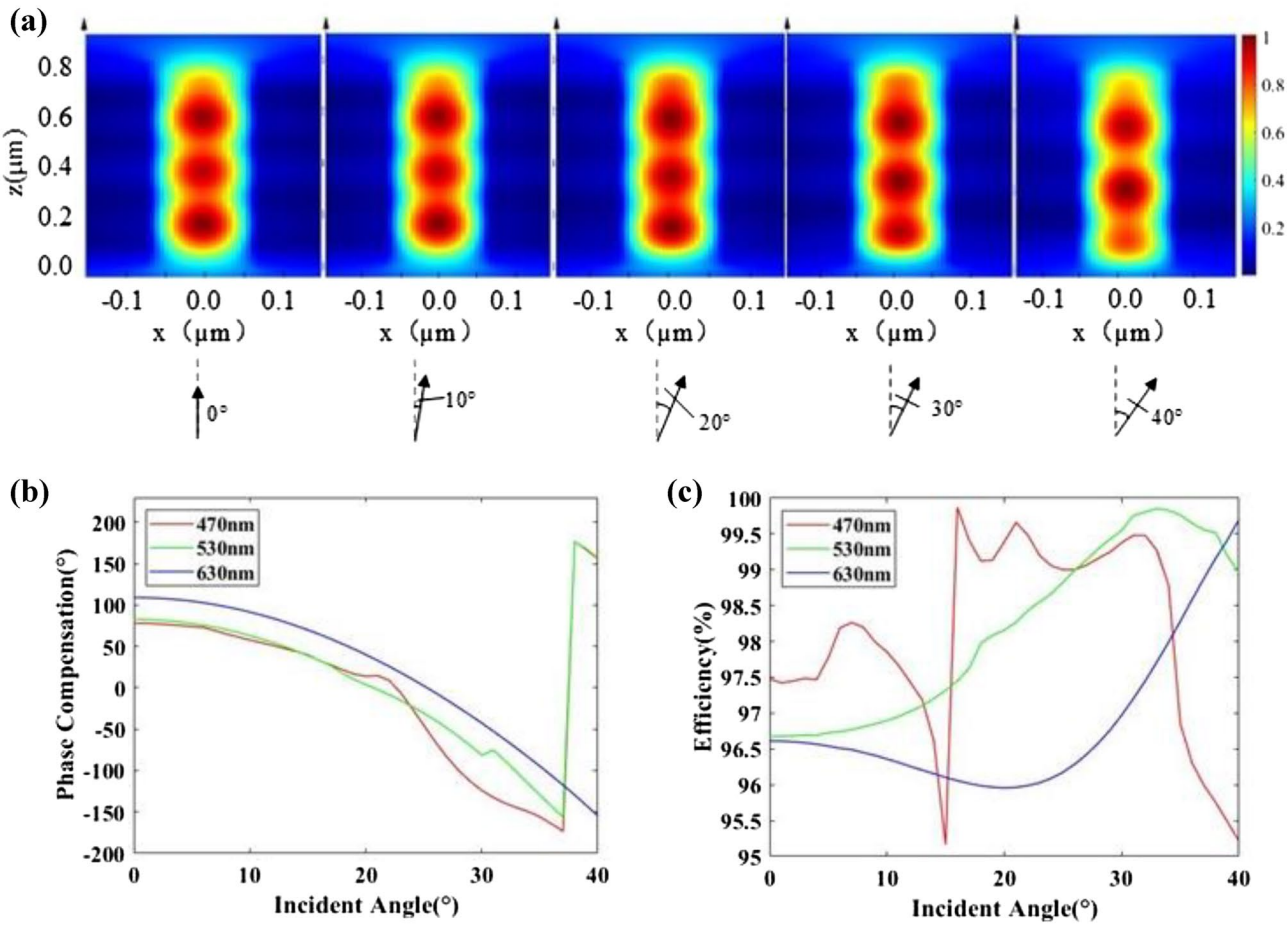
(**a**) Magnetic field intensity distribution under different incident angle. (**b**,**c**) Phase compensation and efficiency with the change of incident angle at the wavelength of 470 nm, 530 nm and 630 nm, respectively.

Take a nanopillar with a diameter of 140 nm as an example, its resonance modes with different incident angle at 530 nm wavelength is simulated under Bloch boundary condition in x and y direction in Lumerical FDTD software as shown in Fig. 3a. The magnetic field intensities of incident angle *θ* = 0°, 10°, 20°, 30° and 40° show that energy is mainly confined inside the high-refractive-index nanopillars, with negligible coupling between the neighboring. Under different incident angles of the target wavelengths, phase compensation and transmission efficiency are shown in Fig. 3b,c, respectively, indicating divergence in the resonance mode. The result illustrates that the double-layers PIAMA used as OFB coupling objectives has the potential of achieving FOV above 80°.

After clarifying the phase modulation requirement of the interface and the characteristics of the nanopillars, the next step is to select the appropriate nanostructures for arrangement. In order to better realize the phase profiles at all target wavelengths, a reference phase mutation is introduced as^35^:

3 $$ \phi_{{{\text{target}}}} \;(x,\;\lambda_{ \, i} ) = \phi_{{{\text{required}}}} \;(x,\;\lambda_{ \, i} ) + C\;(\lambda_{ \, i} ),\quad i = \, 1,\;2, \ldots \;{\text{m,}} $$

where *m* is the number of target wavelengths. The reference phase *C*(*λ*_*i*_) is only dependent on wavelength and has no influence on light modulation. It can be optimized with a powerful optimization algorithm, such as the genetic algorithm and particle swarm algorithm^36^.

To quantify the results of the design, root-mean-square error (RMSE) is employed to calculate the absolute difference between the target phase profile and actual phase profile under

4 $$ RMSE\;(\lambda_{d} ) = \sqrt {{{\sum\limits_{x = 1}^{N} {[\phi_{{{\text{target}}}} \;(x,\;\lambda_{d} ) - \phi_{{{\text{actual}}}} \;(x,\;\lambda_{d} )} ]^{2} } \mathord{\left/ {\vphantom {{\sum\limits_{x = 1}^{N} {[\phi_{{{\text{target}}}} \;(x,\;\lambda_{d} ) - \phi_{{{\text{actual}}}} \;(x,\;\lambda_{d} )} ]^{2} } N}} \right. \kern-\nulldelimiterspace} N}} , $$

where *N* is the number of nanostructures. A higher RMSE means that it is difficult to choose perfect nanostructures in the database to simultaneously satisfy the target phase mutation for distinct wavelengths. Therefore, for a lower RMSE, a straightforward way is to enlarge the database of phase compensation generated by the nanostructures, such as increasing design freedom by adding two or more nanopillars with distinct geometric parameters of nanopillars.

### The design of the focusing layer

In order to concentrate the light energy of each incident direction to an image point at the end face of OFB, the second layer of PIAMA is designed as a polarization-independent achromatic lens at target wavelengths. In addition, the numerical aperture of metalens needs to be less than or equal to the numerical aperture of the OFB.

The design method for the focusing layer is similar to that for the deflecting layer. First, it is necessary to determine the phase profile that the achromatic metalens needs to satisfy under the different wavelengths^37^, as shown in Eq. (5) of

5 $$ \phi \;(x,\;y,\;\lambda_{d} ) = - \frac{2\pi }{{\lambda_{d} }}\left( {\sqrt {x^{2} + y^{2} + f^{2} } - f} \right). $$

Here, in order to match the NA and size of the fibers, the diameter *D* and focal length *f* is set as 30 µm and 50 µm, respectively, and *x* and *y* describe the position of the center of the nanostructures in the Cartesian coordinate system. Since the light from different incident angles is perpendicular to the second layer of PIAMA, there is no need to consider angular dispersion of nanopillars. What’s more, another advantage of this design method is that each unit in the second layer of PIAMA have the same constitution, so the coupling objective can achieve zero telecentricity in image space theoretically.

**Figure 4:**
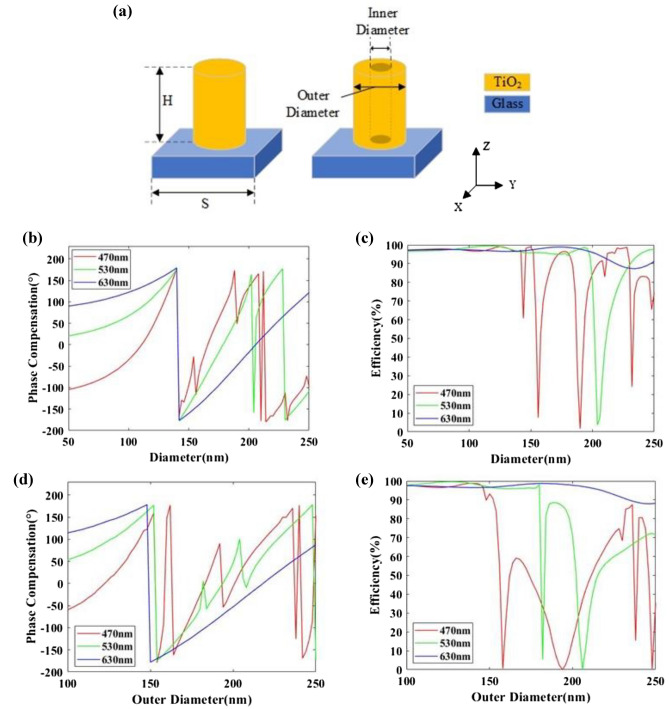
(**a**) Schematic of the nanostructures in focusing layer. The TiO_2_ nanopillar is placed on a glass substrate. (**b**,**c**) Phase compensation and efficiency with the change of diameter of cylindroid nanopillars at the wavelengths of 470 nm, 530 nm and 630 nm, respectively. (**d**,**e**) Phase compensation and efficiency with the change of outer diameter and the inner diameter of 50 nm of annular nanopillars at the wavelengths of 470 nm, 530 nm and 630 nm, respectively.

We still choose isotropic cylindroid and annular nanopillars, which are polarization independent, with the incident electromagnetic waves entering into the nanopillars from the silica substrate as shown in Fig. 4a. The electromagnetic response of the nanopillars is simulated by Lumerical FDTD software and the results of phase compensation and transmission efficiency are shown in Fig. 4b–e. After sweeping nanopillars of different structural parameters, a database of phase compensation and transmission efficiency can be created. Then, the particle swarm algorithm is adopted to optimize *C*(*λ*_*i*_) in Eq. (4) with the minimum RMSE as the goal and the most suitable nanopillars is selected to realize the target phase profile of the wavelengths of 470 nm, 530 nm and 630 nm.

## Results and discussions

### Results of deflecting layer

**Figure 5:**
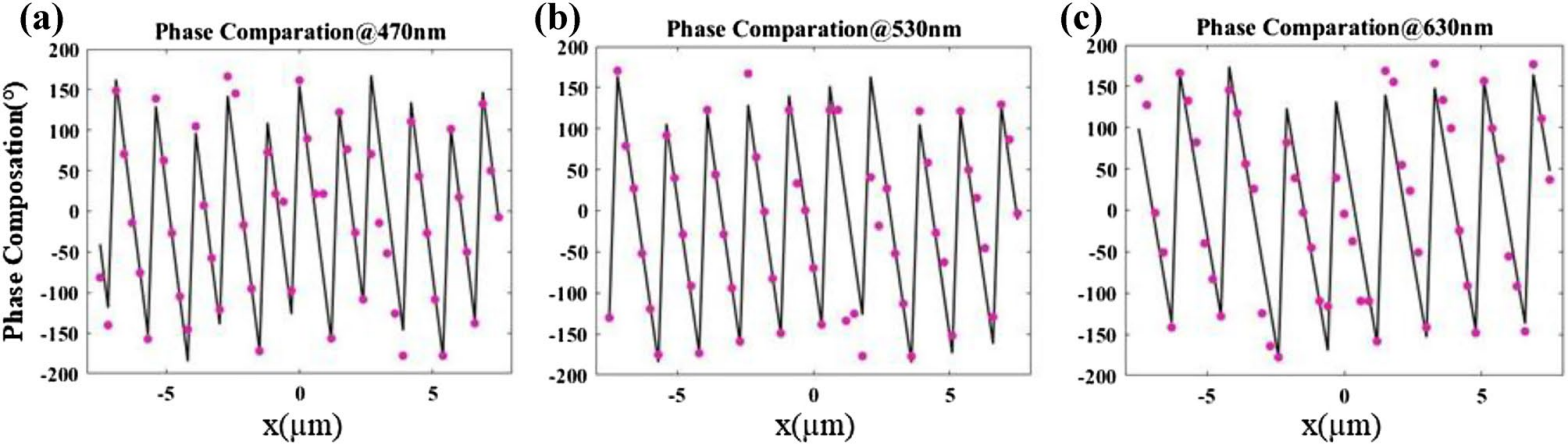
(**a**–**c**) Comparisons of the target phase profiles (solid lines) and actual phase profiles (dot lines) of the deflecting layer at the wavelengths of 470 nm, 530 nm and 630 nm, respectively.

To verify our method and design, the arranged deflecting layer with an incident angle of 20° are numerically simulated using Lumerical FDTD Solutions with the Bloch boundary condition in y direction and perfectly matched layer (PML) boundary condition in x and z direction. According to Eq. (3), the particle swarm algorithm is used to optimize the *C*(*λ*_*i*_) with the lowest sum of the RMSEs of target wavelengths as the evaluation function. Figure 5 shows the comparisons of the target phase profiles (lines) and actual phase profiles (dots) after the optimization procedure.

**Figure 6:**
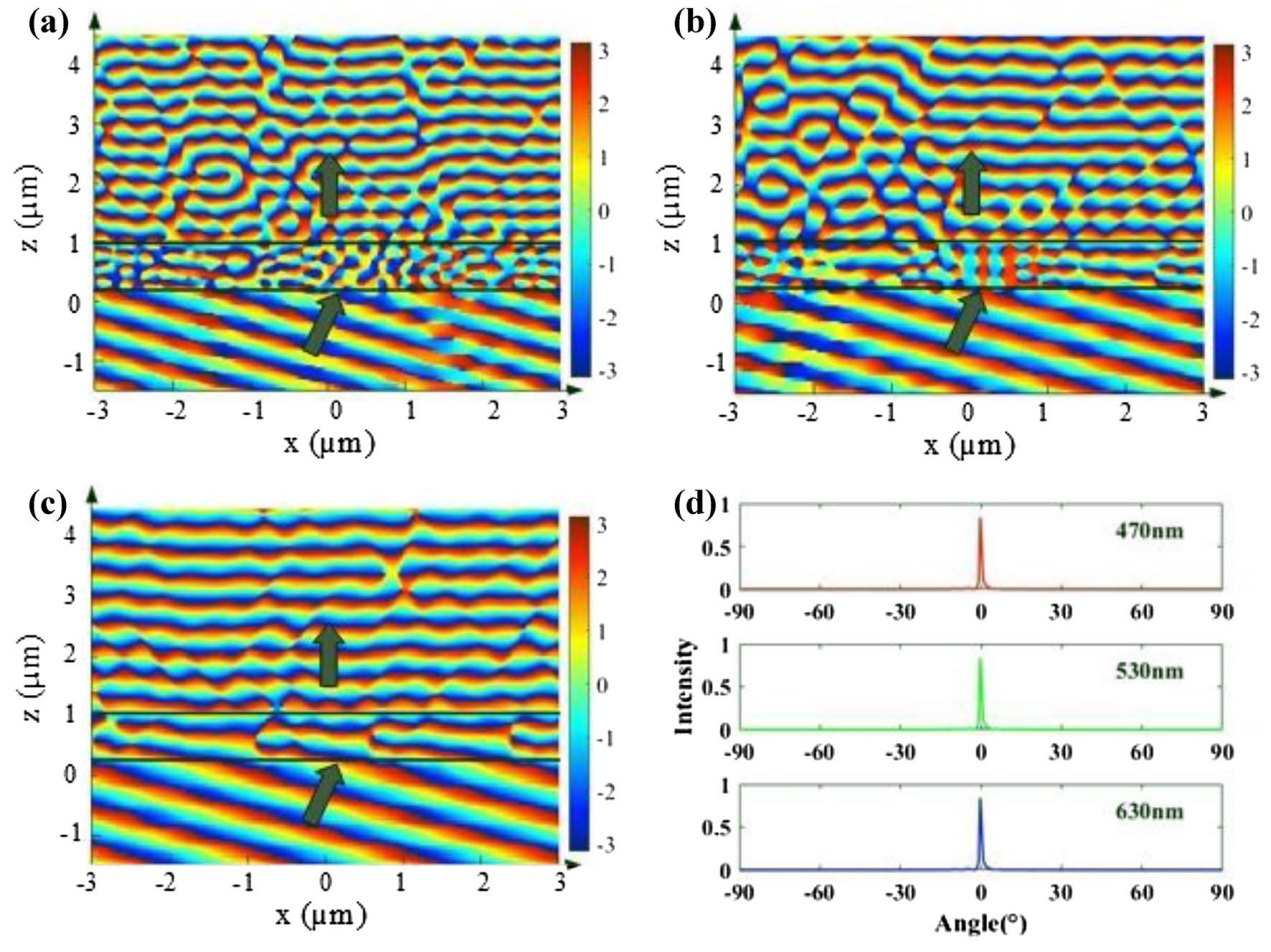
(**a**–**c**) Part of simulated phase distribution of integrated deflecting layer at the wavelengths of 470 nm, 530 nm and 630 nm, respectively. (**d**) Simulated far-field refraction angles at different working wavelengths under 20° incidence condition.

After selecting and arranging suitable nanopillars, some characteristics of the deflecting layer can be received through numerical simulation calculation. As shown in Fig. 6a–c, the phase distribution of x–z plane at the wavelengths of 470 nm, 530 nm and 630 nm indicate that the isophase plane of the refractive electric field is almost parallel to the interface. Because the RMSE between the target phase and the actual phase is 34.04°, 37.45° and 42.64° at the wavelengths of 470 nm, 530 nm and 630 nm, respectively, and the resonance of electromagnetic waves of different frequencies differs inside and between the nanopillars. In addition, scattered light is caused slightly at other angles, which can be discovered more obviously in Fig. 6a than in Fig. 6c. Although the RMSE of 470 nm is smaller than that of 630 nm, the result is a little worse, which is mainly due to the higher absorption rate of TiO_2_ at 470 nm wavelength. Moreover, the far-field intensity distribution map of the three target wavelengths is calculated as shown in Fig. 6d, which expounds that most of the refractive energy concentrates at 0°, and the transmittance efficiency of the first layer of PIAMA is 73.8% at 470 nm, 77.3% at 530 nm and 89.4% at 630 nm.

### Results of focusing layer

**Figure 7:**
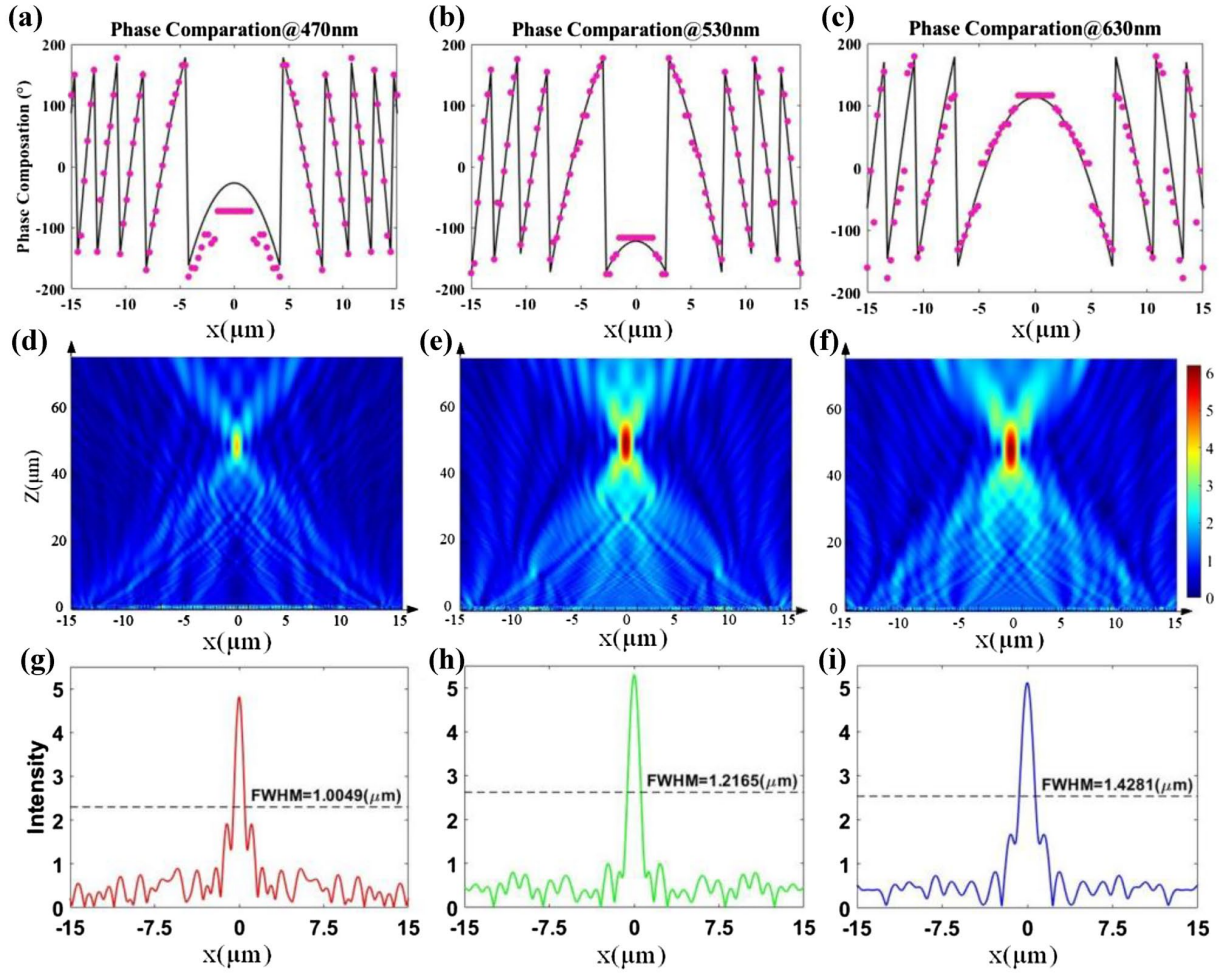
(**a**–**c**) Comparisons of the target phase profiles (solid lines) and actual phase profiles (dot lines) of the focusing layer at the wavelengths of 470 nm, 530 nm and 630 nm, respectively. (**d**–**f**) Simulated electric field intensity distribution of meridional plane at the wavelengths of 470 nm, 530 nm and 630 nm, respectively. (**g**–**i**) Simulated electric field intensity distribution of focal plane at the wavelengths of 470 nm, 530 nm and 630 nm, respectively.

Similar to the design method of deflecting layer, we optimize the target phase profile according to Eq. (3) by using particle swarm optimization as well and pick the suitable nanopillars for smallest RMSE. The comparations between the target and actual phase profiles are shown in Fig. 7a–c, with the RMSEs of 14.05°, 18.93° and 32.75° at the wavelengths of 470 nm, 530 nm and 630 nm, respectively. The integrated focusing layer is numerically simulated by Lumerical FDTD software with the period boundary conditions in y direction and PML boundary conditions in x and z direction.

The electric field intensity distributions on the meridional plane of the three target wavelengths are shown in the Fig. 7d–f. The simulated results show that the electric fields all focus at about 50 µm agree well with our designs. In addition, we place a frequency-domain field and power monitor at the focal plane, and the electric field intensity is shown in the Fig. 7g–i. The full-widths at half-maximum (FWHMs) of the focusing spots along the horizontal direction are 1.0049 µm, 1.2165 µm and 1.4281 µm, respectively, which all approach the diffraction limit. The transmittance efficiencies of the second layer are 63.94%, 74.01% and 88.22%, and the focus efficiencies are 50.68%, 50.70% and 50.63% at the wavelengths of 470 nm, 530 nm and 630 nm, respectively. From the above results it can be seen that the focusing efficiency at 470 nm is worse than that at 530 nm and 630 nm, which should be caused by the slightly larger imaginary part of the refractive index of the TiO_2_ material at 470 nm.

### Results of double-layers metasurface

**Figure 8:**
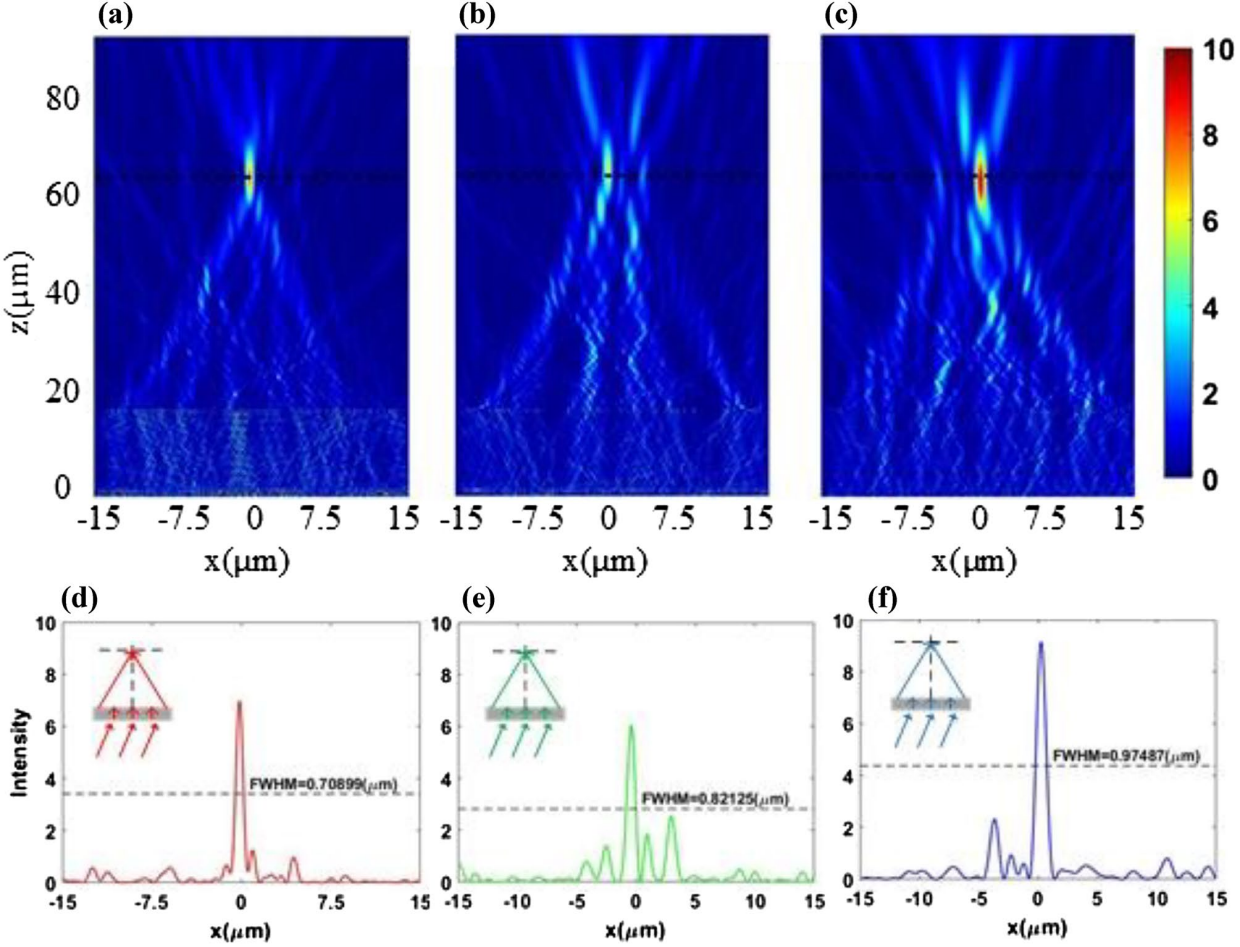
(**a**–**c**) Simulated electric field intensity distribution of double-layers metasurface at the wavelengths of 470 nm, 530 nm and 630 nm, respectively. (**d**–**f**) Simulated electric field intensity distribution of focal plane at the wavelengths of 470 nm, 530 nm and 630 nm, respectively.

After obtaining the numerical verification results of each layer of metasurface, the TiO_2_ nanopillars are arranged on both sides of the silica substrate with the thickness of 15 µm at incident angle of 20°. The two layers of PIAMA have different functions that do not interfere with each other. So, the thickness of substrate between the two layers should be much larger than target wavelengths without near field effect. Besides, the thickness of substrate does not affect the function of PIAMA because the electromagnetic waves transmit perpendicularly to interface. Although the thickness of the substrate may be on the order of millimeters in practice, limited by calculation time and amount in Lumerical FDTD software, we set the thickness of the substrate to 15 µm, which is much larger than the wavelength. The electric field intensity distributions at the wavelengths of 470 nm, 530 nm and 630 nm are shown in the Fig. 8a–c, respectively, indicating that the focal spots of three wavelengths are almost at the same focal plane with a slightly deviation of the center, which is mainly caused by the difference between actual phase compensation of the nanopillars and the design of the target phase profile in the first layer metasurface. If there are more suitable nanostructures which can provide closer phase compensation to the target under the three wavelengths with more freedom in geometric parameters, it is possible to get lower RMSEs and achieve better achromatic effect. In addition, we calculate the FWHMs of the focusing spots as shown in Fig. 8d–f, which are 0.71 µm, 0.82 µm and 0.97 µm and the positions of focusing spots along x axis are − 0.20 µm, − 0.43 µm and 0.23 µm at the wavelengths of 470 nm, 530 nm and 630 nm, respectively. The transmittance efficiencies of the double-layers metasurface are 46.1%, 59.8% and 76.5%, and the focus efficiencies are 41.94%, 31.16% and 44.23%.

## Conclusion

In summary, a double-layer polarization-independent and achromatic metasurface array used for OFB coupling in microendoscope is proposed. Our PIAMA takes example by the structure of insect apposition compound eyes by etching specially designed isotropic circular or annular TiO_2_ nanopillars on two sides of a silica substrate, and each unit in double-layer PIAMA achieves the function of focusing incident light at different incident directions onto the end face of the OFB. Angular dispersion of nanopillars is considered in the first layer, which deflects the incident light without chromatic aberration so that light can enters exits the second layer vertically. The second layer is designed as a metalens that eliminates spherical and chromatic aberrations. We validate our design method by using a unit aiming for incident angle of 20°, obtaining a near-diffraction-limited spot at a focal plane which is 50 μm away from PIAMA. Overall, our work demonstrates that the employment of compact and ultrathin metasurface array which meets the requirement of wide FOV, achromatic aberrations and high telecentricity in image space, is free from bulky size and costly designs compared to the refractive lenses or GRIN lens in microendoscope probe. Although further work is required to verify the performance and feasibility of our double-layers PIAMA for clinical application, it provides a more efficient alternative for the miniaturization of bio-optical.

## Data Availability

All data generated or analyzed during this study are included in this published article and available from the corresponding author on reasonable request.
